# Financial incentives and purchase restrictions in a food benefit program affect the types of foods and beverages purchased: results from a randomized trial

**DOI:** 10.1186/s12966-017-0585-9

**Published:** 2017-09-16

**Authors:** Simone A. French, Sarah A. Rydell, Nathan R. Mitchell, J. Michael Oakes, Brian Elbel, Lisa Harnack

**Affiliations:** 1 Division of Epidemiology and Community Health, University of Minnesota School of Public Health, 1300 S. 2nd Street, Suite 300, 55454, Minneapolis, MN USA; 2 Department of Population Health, School of Medicine, New York University, New York, NY 10016 USA

**Keywords:** SNAP (supplemental nutrition assistance program), Low income, Food purchases, Financial incentives, Restrictions

## Abstract

**Background:**

This research evaluated the effects of financial incentives and purchase restrictions on food purchasing in a food benefit program for low income people.

**Methods:**

Participants (n=279) were randomized to groups: 1) Incentive- 30% financial incentive for fruits and vegetables purchased with food benefits; 2) Restriction- no purchase of sugar-sweetened beverages, sweet baked goods, or candies with food benefits; 3) Incentive plus Restriction; or 4) Control- no incentive or restrictions. Participants received a study-specific debit card where funds were added monthly for 12-weeks. Food purchase receipts were collected over 16 weeks. Total dollars spent on grocery purchases and by targeted food categories were computed from receipts. Group differences were examined using general linear models.

**Results:**

Weekly purchases of fruit significantly increased in the Incentive plus Restriction ($4.8) compared to the Restriction ($1.7) and Control ($2.1) groups (p <.01). Sugar-sweetened beverage purchases significantly decreased in the Incentive plus Restriction

(−$0.8 per week) and Restriction ($-1.4 per week) groups compared to the Control group (+$1.5; p< .0001). Sweet baked goods purchases significantly decreased in the Restriction (−$0.70 per week) compared to the Control group (+$0.82 per week; p < .01).

**Conclusions:**

Paired financial incentives and restrictions on foods and beverages purchased with food program funds may support more healthful food purchases compared to no incentives or restrictions.

**Clinical trial registration:**

Clinicaltrials.gov Identifier: NCT02643576.

## Background

Poor dietary quality, including low fruit and vegetable and high sugar-sweetened beverage intake, is especially prevalent among lower income Americans [1, 2]. National data show that people with lower incomes consume fewer fruits and vegetables and more sugar-sweetened beverages compared with higher income people [3–6]. Poor diet quality is believed to be an important contributor to the high prevalence of obesity and diet-related chronic diseases observed among lower income people [3, 7, 8].

Food purchasing behavior has received little research attention as an intervention target to improve diet quality among low-income families [9–12]. Food purchasing behavior is a potentially strong mediator between income and diet quality, since foods present in the home directly influence individual food choices and eating behaviors [13, 14]. Randomized trials to evaluate interventions to improve the nutritional quality of foods and beverages purchased among lower-income people are few. The USDA Healthy Incentives Pilot (HIP), a randomized trial among low-income Supplemental Nutrition Assistance Program [SNAP; federal food stamp program] participants, provided financial incentives to SNAP-enrolled households for the purchase of fruits and vegetables for a one-year period [11]. The results showed that those receiving financial incentives for fruit and vegetable purchases purchased and consumed more fruits and vegetables than those in the comparison group.

High intake of foods high in added sugars, such as sugar-sweetened beverages, candy and sweet baked goods, contributes to poor dietary quality and high obesity risk [15]. It has been suggested that federal food programs such as SNAP restrict the purchase with program funds of foods high in added sugars by enrolled participants [16, 17]. Currently, the only restrictions on SNAP benefit use is on the purchase of alcoholic beverages, restaurant food, or dietary supplements [18]. Restriction of the use of program funds for the purchase of high-added sugar items is hypothesized to reduce the purchase of these items, and thereby reduce program participant intake of these “empty calorie” foods and beverages. However, it is possible that restrictions would have no effect on food purchasing or consumption since out-of-pocket funds may be used to purchase prohibited foods. To our knowledge, no randomized trial has been conducted to examine the effects of restrictions on the purchase of certain food and beverage items.

The present study examined the effects of the provision of financial incentives for the purchase of fruits and vegetables, restriction of the purchase of sugar-sweetened beverages, candy, and sweet baked goods, or both, on food purchases among lower income adults. It was hypothesized that restrictions on the purchase of targeted foods and beverages would result in decreases in the purchase of those items, and that incentives for the purchase of fruits and vegetables would result in increases in the purchase of fruits and vegetables, compared with no incentives for, or restrictions on, foods and beverages purchased. Results for the effects of these interventions on dietary intake are reported elsewhere [19].

## Methods

### Study overview

Data for the present study were collected as part of a randomized trial that enrolled lower-income adults not currently participating in SNAP [19]. Participants were randomized to one of four groups: 1) Incentive- 30% financial incentive for fruits and vegetables purchased with food benefits; 2) Restriction- no purchase of sugar-sweetened beverages, sweet baked goods, or candies with food benefits; 3) Incentive plus Restriction; or 4) Control- no incentive or restrictions on foods purchased with food benefits. Participants in all groups were given a study-specific debit card where funds were added every four weeks for a 12-week period. Food purchases were measured using food and beverage receipts collected over a 4-week baseline period and throughout the 12-week experimental period. Details are published elsewhere [19].

### Eligibility criteria and recruitment

Households were recruited between August 2013 and May 2015 in the Minneapolis-St. Paul, Minnesota metropolitan area using fliers placed in community locations in high-poverty neighborhoods and through organizations that serve low-income households. Individual level measures (e.g. demographic information, height, weight, dietary recalls) were collected from the adult in the household most responsible for food shopping. Study eligibility criteria were established with the aim of recruiting adults in households that were near eligible for SNAP or eligible for SNAP but not currently enrolled. Eligibility criteria were: 1) not currently enrolled in SNAP; 2) household income ≤200% of the federal poverty rate or participating in a government program, such as the Diversionary Work Program, which automatically qualifies households for SNAP in Minnesota; and 3) the adult in the household who is primarily responsible for food shopping is able to read and speak English and is willing to participate. Other SNAP eligibility criteria, such as an asset test, or US citizenship, were not applied.

### Experimental procedures

Those who completed baseline measures and remained eligible (no SNAP EBT card usage detected on baseline food purchase receipts) were randomized to one of the four food benefit experimental conditions: Incentive, Restriction, Incentives plus Restriction, or Control.

Participants in all conditions received financial assistance for the purchase of grocery food and beverages for a 12-week period. The amount of financial assistance was equal to the average benefit amount per household size provided by the federal food assistance program (also known as SNAP) in Hennepin/Ramsey counties in Minnesota in June 2013 ($152 monthly for household of 1; $277 monthly for household of 2; $401 monthly for household of 3, etc) [15].

The grocery financial assistance was delivered to participants using a study-specific debit card, similar to that used in the SNAP federal food program. Funds were added to the participant’s card every four weeks. Participants in all groups were instructed to follow the federal SNAP guidelines for eligible food and beverage purchases when using their study food purchasing cards (e.g., no purchase of alcoholic beverages, hot prepared foods or restaurant foods: see Table 1 for details). Those in the Restriction and Incentive plus Restriction groups were also told they could not purchase sugar-sweetened beverages, candy, or sweet baked goods using the study-provided debit card. Those in the Incentive and Incentive plus Restriction groups were told that for every dollar from their card spent on eligible fruits or vegetables, they would receive $0.30 cash credited back onto their card. Fruits and vegetables not eligible for the incentive included white potatoes; 100% fruit juices and fruit drinks; pickled vegetables; and fruits and vegetables with sauces or sugar added. The incentive for purchasing eligible fruits and vegetables was provided on a weekly basis, with the incentive amount added to the debit card and a text or email sent to the participant notifying him/her of the incentive amount added to their card. The incentive amount was determined by reviewing food purchase receipts submitted by participants each week.

**Table 1:** Description of Experimental Conditions

Food Purchase Rules	Experimental Condition			
	Incentive	Restriction	Incentive plus Restriction	Control
Not allowed to purchase alcoholic beverages, restaurant foods, and dietary supplements with debit card (same exclusion criteria as SNAP)	x	x	x	x
Not allowed to purchase sugar sweetened beverages (water-based beverages with added sugar such as soft drinks, fruit drinks, energy drinks, and sports drinks), candy (all types), and prepared sweet baked goods (e.g. pies, cakes, cookies, donuts) with debit card		x	x	
30% incentive on eligible^a^ fruits and vegetables; Incentive amount calculated weekly from food purchase receipts and added to debit card. Text/email sent notifying participant of amount added as incentive.	x		x	

^a^Fruits and vegetables not eligible for 30% incentive include fruit juices; fruits canned, frozen or dried with sugar/syrup; vegetables canned or frozen with a sauce; pickled vegetables; and white potatoes

To encourage compliance in use of the study debit card in accord with group-specific spending rules, appropriate use was explained verbally and in writing. Feedback was provided when error in use of the card was detected. Repeated or obvious misuse of the card resulted in termination of the debit card (removal of existing funds with no additional funds provided). Compliance was monitored on an ongoing basis using food purchase receipts submitted by participants in conjunction with transaction information available through the bank that administered the debit cards.

### Measures

Baseline measures were collected over a four-week period, and included collection of three telephone administered 24-h dietary recalls; height and weight measured by trained research staff at the University of Minnesota; self-reported demographic information; and four weeks of household food purchase receipts. Household food purchase receipts were collected continuously throughout the 12-week experimental period. Consistent with an intent-to-treat study approach, follow-up measures were sought from participants who were terminated from receiving debit card funds due to non-compliance with their group-specific spending rules. Incentives (gift cards to a discount retailer) were provided for completion of study evaluation measures, including $30 for every four weeks of food purchase receipt collection.

#### Food purchases

Food purchases were measured using food and beverage receipts. Participants were instructed, trained, and provided with feedback on the receipt collection protocol by study staff [13, 14, 20]. Participants were instructed to collect receipts from every food and beverage purchased from any source, including grocery stores, gas stations, discount stores, farmers markets, food co-ops, restaurants and coffee shops. They were also instructed to collect receipts from household family members for any foods and beverages purchased.

For all non-restaurant food purchase receipts (subsequently referred to as ‘grocery receipts’), participants were instructed to annotate the receipt to provide missing food detail. For example, if a line item on a receipt was ‘produce’ the participant was instructed to record on the receipt a more complete description of the food item (e.g. ‘bananas’).

Participants were trained to complete a missing receipt form for every food and beverage purchase for which they did not have a receipt. Food and beverages purchased were itemized on the missing receipt form, and the amount spent for each item, the store name and location and date were recorded. Missing receipt forms were returned to study staff with other receipts.

Food and beverage purchases on the receipts were coded by trained study staff according to a standardized protocol [13, 14, 20]. For all receipts (restaurant and grocery receipts) the total dollar amount spent on foods and beverages was tallied. Non-food items and taxes were not included in the computation of total food spending. For grocery receipts, food and beverage items on the receipt were coded into a variety of food categories, including: 1) fruit; 2) vegetable; 3) sugar-sweetened beverages; 4) sweet baked goods; 5) candy and 6) savory snacks. Classification criteria for the fruit, vegetable, sugar-sweetened beverages, sweet baked goods, and candy categories corresponded with the incentive and restriction criteria for the experiment (e.g. potatoes were not coded as a vegetable). For each food category, the total dollar amount spent for the food was coded.

Variables computed from the receipt data included in the present paper are as follows: average weekly grocery spending (dollars/week) at baseline and follow-up on: 1) fruits (without 100% juice and without fried fruits); 2) vegetables (without fried vegetables and without white potatoes); 3) sugar-sweetened beverages (without 100% juice); 5) sweet baked goods; and 6) candy. Average total weekly spending at baseline and follow-up on restaurant and grocery purchases were also calculated. Total average weekly grocery spending was computed by summing across all food categories. Restaurant purchases were recorded as a separate category and only included total dollars spent. Food and beverages were not individually coded from restaurant receipts and are not included in the computation of dollars spent on specific food and beverage categories.

The first four weeks of food purchase receipts were collected prior to randomization and served as the baseline food purchase data. The 12 weeks of receipts collected after randomization served as the follow-up food purchase data. Weeks were averaged to estimate weekly spending on food and beverage categories and total weekly grocery spending at baseline (weeks 1–4 average) and follow-up (weeks 5–16 average).

#### Demographic and other survey measures

Demographic variables were measured using participant self-reports on a survey administered at baseline. Variables included gender, age, marital status, education, household income, and household size. Participants were also asked about their current and past participation in food assistance programs and household food security [21] was assessed.

### Statistical analysis

**Fig. 1:**
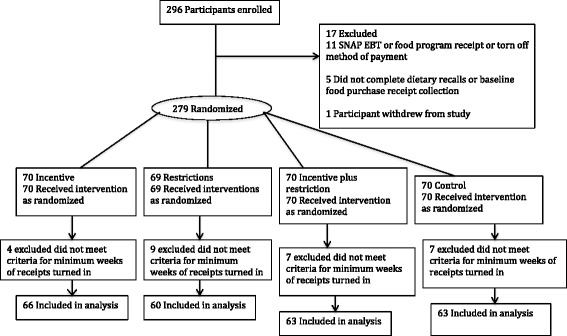
Consort Diagram

All statistical analyses were conducted using SAS statistical software (version 9.4; SAS Institute Inc., Cary NC). The analytic sample was restricted those with ≥3 weeks of food purchase receipts at baseline and ≥9 weeks during the follow-up period (*n* = 252 of 279 randomized: see Fig. 1). The reason for the minimum number of weeks of receipt criterion was due to concerns that only two weeks of receipts would not validly represent usual food purchasing in this low-income sample [13, 14, 20]. The number of participants excluded from the analysis due to not meeting the minimum number of receipts did not differ meaningfully by treatment group assignment (incentive: *n* = 4; restriction: *n* = 9; incentive plus restriction: *n* = 7; control: n = 7).

Group differences in change in food purchasing were examined using general linear regression. Separate models were run for each of the dependent variables: change (follow-up average weekly spending - baseline average weekly spending) in spending on fruits, vegetables, sugar-sweetened beverages, sweet baked goods, candy, total grocery spending and total restaurant spending. Results were considered statistically significant where *p* < 0.05. Covariates were not included in the models because groups were similar with respect to potentially confounding factors such as household size and food security status. Results are presented for Type I error rates unadjusted for multiple testing because all hypotheses were identified a priori and maintain the nominal error rate for the family of tests. In addition, results are presented for all outcomes examined (reporting is not selective).

## Results

### Demographic information

Demographic data are shown in Table 2. Average age of participants was 45 yrs., most participants were women (81%), about half were African American (52%), most had less than a college level education (81%), less than half were married or living with a partner (29%) and 82% were overweight or obese. Thirty-one percent of households reported annual income of $14,999 or less. Eighty-eight percent had low or very low food security.

**Table 2:** Demographic Characteristics at Baseline by Experimental Group: Lower-Income Adults Enrolled in the Grocery Assistance Program Study (*n* = 252)

	Total	Incentive	Restriction	Incentive plus Restriction	Control	*p*
Age in years mean (se)	45.0 (1.6)	42.8 (1.6)	45.3 (1.7)	47.4 (1.6)	44.6 (1.6)	.24
Race % (n)						.38
White	31.1 (78)	27.3 (18)	23.7 (14)	41.3 (26)	31.7 (20)	
African American	51.6 (130)	53.0 (35)	60.0 (36)	42.9 (27)	50.8 (32)	
Biracial	12.3 (31)	13.6 (9)	8.3 (5)	11.1 (7)	15.9 (10)	
Others	5.2 (13)	6.1 (4)	8.3 (5)	4.8 (3)	1.6 (1)	
Ethnicity						
Latino/Hispanic % (n)	3.6 (9)	4.5 (3)	6.7 (4)	3.2 (2)	0 (0)	
Marital status % (n)						.14
Married/marriage-like relationship	28.7 (180)	19.7 (13)	25.0 (15)	33.9 (21)	36.5 (23)	
Single	43.0 (108)	30.6 (33)	28.7 (31)	23.2 (25)	17.6 (19)	
Divorced/separated	28.3 (251)	28.2 (20)	19.7914)	22.5 (16)	29.6 (21)	
Female % (n)	81.4 (205)	84.9 (56)	80.0 (48)	79.4 (50)	81.0 (51)	.86
Education % (n)						.25
High school graduate or less	27.4 (69)	24.6 (17)	30.4 (21)	23.2 (16)	21.7 (15)	
Some college	53.2 (134)	30.6 (41)	22.4 (30)	23.1 (31)	23.9 (32)	
College degree or more education	19.4 (49)	16.3 (8)	18.4 (9)	32.7 (16)	32.7 (16)	
Body weight category						.77
% (n) normal weight [BMI ≤ 25 kg/m^2^]	17.4 (42)	18.2 (12)	15.3 (9)	20.7 (12)	15.5 (9)	
overweight [25 < BMI ≤ 27 kg/m^2^]	24.9 (60)	21.2 (14)	30.5 (18)	27.6 (16)	20.7 (12)	
obese [BMI > 27 kg/m^2^]	57.7 (139)	60.6 (40)	54.2 (32)	51.7 (30)	63.8 (37)	
Income % (n)						
< $15,000	30.6 (77)	22.7 (15)	36.7 (22)	31.7 (20)	31.7 (97)	.63
$15,000- < $35,000	43.7 (110)	47.0 (31)	40.0 (24)	46.0 (29)	41.3 (26)	
$35,000–$75,000	18.3 (46)	22.7 (15)	11.7 (7)	17.5 (11)	20.6 (13)	
Household Size % (n)						
1 Study Benefit $139/mo	23.8 (60)	21.2 (14)	25.0 (15)	30.1 (19)	19.1 (12)	.54
2 Study Benefit $233/mo	21.8 (55)	16.7 (11)	25.0 (15)	20.6 (13)	25.4 (16)	
3 Study Benefit $350/mo	22.6 (57)	24.2 (16)	15.0 (9)	19.1 (12)	31.8 (20)	
4 Study Benefit $421/mo	14.3 (36)	16.7 (11)	13.3 (8)	15.9 (10)	11.1 (7)	
≥ 5 Study Benefit $493/mo	17.5 (44)	21.2 (14)	21.7 (13)	14.3 (9)	12.7 (8)	
Food Security % (n)						
Very Low	45.2 (114)	42.4 (28)	46.7 (28)	47.6 (28)	44.4 (28)	.24
Low	34.5 (87)	45.5 (30)	33.3 (20)	31.8 (20)	27.0 (17)	
High or marginal	20.2 (51)	12.1 (8)	20.0 (12)	20.6 (13)	28.6 (18)	

### Change in food purchasing by group

Table 3 shows baseline, follow-up, and change in food and beverage purchases for grocery, restaurant, and specific food and beverage categories by experimental group. Categories do not sum to the total grocery spending because only categories that are the focus of the present analysis are included in this report. At baseline, average weekly grocery purchases per household ranged approximately $65–$72 across experimental groups. Average weekly restaurant purchases ranged about $22–$30 across groups. Grocery purchases for fruits averaged about $4 per week and vegetables about $4–5 per week. Sugar-sweetened beverage purchases averaged about $3–4 per week. Sweet baked goods purchases each averaged about $2–3 per week, and candy purchases averaged about $1–2 per week.

**Table 3:** Grocery, Restaurant and Targeted Food Purchases (Average Dollars per Week) by Experimental Condition (n = 252)(Mean, Standard Error, and 95% Confidence Interval)

	Total mean (SE)	Incentive mean (SE) 95% CI^a^	Restriction mean (SE) 95% CI	Incentive Plus Restriction mean (SE) 95% CI	Control mean (SE) 95% CI	*p*
N	252	66	60	63	63	
Dollars spent (average per week)						
Groceries						
Baseline	68.5 (3.4)	72.7 (6.7) 59.5, 85.8	66.1 (7.0) 52.4, 79.9	64.9 (6.8) 51.5, 78.3	70.1 (6.8) 56.6, 83.5	
Follow up	101.8 (2.8)	104.2 (5.5) 93.4, 115.0	94.6 (5.8) 83.2, 105.9	102.6 (5.6) 91.5, 113.7	105.3 (5.6) 94.2, 116.4	
Change^b^	33.3 (2.4)	31.5 (4.7) 22.2, 40.9	28.5 (5.0) 18.7, 38.3	37.7 (4.8) 28.2, 47.3	35.2 (4.8) 25.7, 44.8	.56
Restaurant						
Baseline	25.1 (1.7)	29.6 (3.4) 22.9, 36.3	23.7 (3.6) 16.7, 30.8	24.7 (3.5) 17.8, 31.6	22.0 (3.5) 15.2, 28.9	
Follow up	18.6 (1.5)	23.2 (2.9) 17.5, 28.9	13.9 (3.0) 7.9, 19.9	19.3 (3.0) 13.4, 25.1	17.6 (3.0) 11.7, 23.4	
Change	−6.5 (1.1)	−6.4 (2.2) −10.8, −2.1	−9.8 (2.3) −14.4, −5.3	−5.4 (2.3) −9.9, −0.98	−4.5 (2.3) −8.9, −0.03	.38
Fruit						
Baseline	4.0 (0.3)	3.6 (0.6) 2.5, 4.8	4.3 (0.6) 3.1, 5.6	3.7 (0.6) 2.5, 4.9	4.2 (0.6) 3.0, 5.4	
Follow up	7.0 (0.4)	7.0 (0.8) 5.4, 8.6	6.0 (0.9) 4.3, 7.7	8.5 (0.9) 6.8, 10.1	6.4 (0.9) 4.7, 8.0	
Change^c^	3.0 (0.3)	3.3 (0.6) 2.1, 4.6	1.7 (0.7) 0.34, 3.0	4.8 (0.7) 3.5, 6.0	2.1 (0.7) 0.86, 3.4	.01
Vegetables						
Baseline	4.2 (0.3)	3.9 (0.6) 2.6, 5.1	4.6 (0.7) 3.3, 5.9	3.7 (0.7) 2.4, 5.0	4.5 (0.7) 3.3, 5.8	
Follow up	7.1 (0.4)	6.7 (0.7) 5.4, 8.1	6.9 (0.7) 5.4, 8.3	7.7 (0.7) 6.3, 9.1	6.9 (0.7) 5.5, 8.4	
Change	2.9 (0.3)	2.9 (0.5) 1.9, 3.9	2.3 (0.6) 1.2, 3.4	4.0 (0.5) 2.9, 5.1	2.4 (0.5) 1.3, 3.5	.10
Sugar Sweetened Beverages						
Baseline	3.4 (0.3)	3.9 (0.5) 2.9, 5.0	3.7 (0.6) 2.6, 4.8	3.1 (0.5) 2.0 4.2	3.0 (0.5) 1.9, 4.1	
Follow up	3.3 (0.2)	4.2 (0.4) 3.3, 5,0	2.3 (0.4) 1.4, 3.2	2.3 (0.4) 1.5, 3.2	4.5 (0.4) 3.6, 5.3	
Change^d^	−0.1 (0.2)	0.2 (0.4) −0.55, 1.03	−1.4 (0.4) −2.2, −0.59	−0.8 (0.4) −1.6, 0.01	1.5 (0.4) 0.69, 2.3	.0001
Sweet Baked Goods						
Baseline	2.4 (0.2)	2.8 (0.4) 2.0, 3.6	2.3 (0.4) 1.5, 3.1	2.0 (0.4) 1.2, 2.8	2.5 (0.4) 1.7, 3.3	
Follow up	2.6 (0.2)	3.6 (0.3) 3.9, 4.2	1.6 (0.3) 0.89, 2.2	1.9 (0.3) 1.2, 2.5	3.3 (0.3) 2.7, 4.0	
Change^e^	0.2 (0.2)	0.8 (0.3) 0.10, 1.5	−0.7 (0.4) −1.4, 0.01	−0.1 (0.4) −0.78, 0.61	0.8 (0.4) 0.13, 1.5	.01
Candy						
Baseline	1.4 (0.1)	1.5 (0.2) 1.0, 2.0	1.3 (0.3) 0.78, 1.8	1.2 (0.3) 0.69, 1.7	1.7 (0.3) 1.3, 2.2	
Follow up	1.5 (0.1)	1.7 (0.2) 1.3, 2.2	1.0 (0.3) 0.52, 1.5	1.3 (0.2) 0.78, 1.7	2.1 (0.2) 1.6, 2.6	
Change	0.1 (0.1)	0.2 (0.2) −0.24, 0.72	−0.3 (0.3) −0.76, 0.23	0.1 (0.3) −0.41, 0.57	0.3 (0.3) −0.16, 0.81	.36

^a^95% Confidence Interval

^b^Change = follow up - baseline. Baseline is the weekly average for weeks 1–4. Follow up is the weekly average for weeks 5–16. Values are unadjusted

^c^Difference in change is significantly different (*p* < .05) between the Incentives Plus Restriction and the Control groups; and between the Restriction and the Incentives Plus Restriction groups

^d^Difference in change is significantly different (*p* < .05) between the Incentives and the Control groups; the Restrictions and the Control groups; the Incentives Plus Restrictions and the Control group; and the Incentives and the Restrictions groups

^e^Difference in change is significantly different (p < .05) between the Incentives and the Restrictions groups; and between the Restrictions and the Control groups

At follow-up, average weekly grocery purchases increased in all groups (+$29 - + $38), with the magnitude of increase similar across conditions. In contrast restaurant purchases decreased in all groups (−$5 to -$10 per week), with the magnitude of decrease similar across conditions.

With respect to changes in purchases for specific categories of food, the increases in the purchase of fruit were significantly greater in the Incentive plus Restriction group (+$4.8 per week) compared to the Control (+$2.1 per week; *p* < .01) group. Change in purchases of vegetables did not significantly differ by group.

Changes in sugar-sweetened beverage purchases in the Incentive plus Restriction

(−$0.80 per week) and Restriction (−$1.4 per week) groups were significantly different compared with change in the Control group (+$1.5 per week; *p* < .0001). Change in sweet baked goods purchases were significantly different in the Restriction (−$0.70 per week) group compared with the Control group (+$.82 per week; p < .01). No significant differences between groups were observed for changes in purchases of candy.

## Discussion

The purpose of the present study was to evaluate whether a food benefit program that incentivizes and/or restricts the purchase of certain foods and beverages influences the purchase of these foods in lower-income households. Results suggest that both incentives and restrictions may change the purchase of some of the targeted foods and beverages.

Participants who received a financial incentive for the purchase of fruits and vegetables (30% of purchase price), in conjunction with restrictions on the purchase of sugar-sweetened beverages, sweet baked goods and candy, increased grocery purchases of fruits (but not vegetables) to a greater extent than those who did not receive a financial incentive. The increase in purchases of fruit in the Incentive plus Restriction group was about double the amount of the groups that received no financial incentive for fruit and vegetable purchases. Changes in purchases of vegetables were not significantly different between groups, and were similar in direction (vegetable spending increased in all groups). These findings are largely consistent with a major community intervention trial conducted by the USDA Food and Nutrition Services that randomized SNAP participants to receive financial incentives for fruit and vegetable purchase (30% of purchase price), or to a no-incentives group [11]. In that study, the incentivized group purchased about a dollar per month more fruits and vegetables than the no-incentive group (according to the individual participant EBT purchase data). Fruit and vegetable purchasing was not examined separately in the USDA study, and thus it is unclear whether spending was higher for both fruits and vegetables or just one of these food categories. These results are also consistent with laboratory experimental studies that financially incentivized the purchase of fruits and vegetables [22, 23]. Although the magnitude of changes in purchasing observed in other studies and in the present study is small at the individual level, it may be significant in terms of the population impact on change in fruits and vegetable purchases [24], and is particularly important because both the SNAP Healthy Incentives Pilot and the present study were conducted in low-income populations, who are at the high risk for low fruit and vegetable intake and poor diet quality [1–6].

It is interesting to note that offering an incentive for the purchase of fruits and vegetables did not appear to shift purchases of foods for which consumption is discouraged [25, 26] (e.g. sugar-sweetened beverages, sweet baked goods and candy). In the present study, no differences were observed between the incentive-only group and the control group for changes in the purchase of sugar-sweetened beverages, sweet baked goods or candy. These findings, and the results of others [25, 26], suggest that offering a financial incentive for the purchase of healthful foods may neither increase nor decrease the purchase of foods high in added sugars.

Restricting the use of food program benefits for purchasing sugar-sweetened beverages, sweet baked goods and candy appeared to be effective in reducing the purchase of sugar-sweetened beverages and sweet baked goods [25, 27–29]. The results suggest that interventions that limit purchases of sugar-sweetened beverages and other empty-calorie foods may be effective in decreasing spending for these foods, and thus may contribute to improvements in dietary quality. The results from the present trial (reported elsewhere; 19) showed that compared to the control group, food purchase incentives and restrictions resulted in significant increases in intake of solid fruit, decreases in intake of sugar-sweetened beverages, sweet baked goods and candy, and higher Healthy Eating Index scores among the adult in the household primarily responsible for food shopping [19]. These findings are informative in the context of the current policy debate regarding whether purchase of these types of foods and beverages should be allowed with federal nutrition program funds, such as SNAP. It is interesting to note that some spending on these categories of foods persisted during the experimental period, indicating that out of pocket funds were used in place of food benefit funds to purchase restricted foods. However, results suggest that out of pocket funds did not fully replace what otherwise may have been spent on these types of foods [26, 30]. Results from the dietary intake data suggest that restrictions and incentives may have important effects not only on intake of these foods, but also on overall diet quality. Since household food purchases affect the foods and beverages available in the home, it is possible that restrictions and incentives may positively improve other household members’ dietary intake. Further research is warranted to explore the potential positive effects of restrictions and incentives on both food purchases and dietary intake of all household members.

The present study had several important strengths, including its randomized design, naturalistic setting, and low-income sample. Limitations include the methodological weaknesses inherent in the receipt collection methodology. No objective measure exists of the true total number of receipts that participants should turn in to the research staff. It is possible that participants may have omitted receipts for small purchases such as a single drink or candy item [13, 14]. Participants may have selectively turned in the receipts for which the study debit card was capable of tracking and omitted other receipts from foods purchased with their own money. By contrast, a strength of the receipt data is its potentially lower reactivity compared with the assessment of individual dietary intake using a verbally reported 24-h dietary intake interview. Food purchase receipts are an objective measure of food purchases, do not rely on participant memory, and may be less affected by social desirability responding. Food purchases and dietary intake also measure different constructs, each distinctly of interest in the context of the evaluation of food program incentives and restrictions. The food purchase receipts reflect household food purchases, which are directly affected by food program policies about food and beverage purchases using program funds, and may influence the dietary intake and quality of all household members. Dietary intake data using dietary recalls reflect the intake of one individual in the household, and are known to be biased by selective underreporting and social desirability. In the present study, the quantities corresponding to the dollar amount spent on each of the incentivized and restricted food and beverage categories was not captured. For example, the quantity of fruit purchased with $5 varies depending on the price of the particular fruit purchased. Our previous work suggests that patterns of results for outcomes of interest related to household food purchases are similar when dollars spent and pounds or ounces purchased are examined [13, 14]. The present research did not examine the household’s marginal propensity to purchase certain foods, such as fruits and vegetables and sugar-sweetened beverages, using program funds versus out-of-pocket funds [26, 30]. This question is being addressed in a separate report using data from the present study. Participants in the study received payments for the evaluation portion of the study in the form of a gift card to a discount retailer (Target). The gift card could be used to purchase personal and household items, including food. It is expected that any potential effects of study data collection payments on food purchases would be equal across all study conditions, since participants were randomized to study conditions and all received identical payments for data collection activities. The enrolled sample was similar but not identical to a SNAP-eligible sample in terms of income and other demographic variables, and this could affect the generalizability of the results reported here. Finally, it would have been useful to continue to follow the study participants over a longer time period after the grocery funding payments ended, to examine the persistence of changes in dietary intake and household food and beverage purchases. Due to limited funding, this was not possible, but is recommended for future research. Considered in total, it is believed that the strengths of the present study’s methodology outweigh its limitations, and that it improves upon other study methodologies available in the literature to date.

## Conclusions

In conclusion, results suggest that financial incentives and restrictions on the types of foods and beverages purchased with food program funds can support more healthful food purchases among lower income people. These findings have important implications for federal food program policies to influence population dietary intake. Future research is needed to examine these interventions in a SNAP-enrolled sample and over a longer time period.
